# Correcting systematic errors in diffraction data with modern scaling algorithms

**DOI:** 10.1107/S2059798323005776

**Published:** 2023-08-16

**Authors:** Luis A. Aldama, Kevin M. Dalton, Doeke R. Hekstra

**Affiliations:** a Department of Molecular and Cellular Biology, Harvard University, Cambridge, Massachusetts, USA; b Biophysics Graduate Program, Harvard University, Cambridge, Massachusetts, USA; c John A. Paulson School of Engineering and Applied Sciences, Harvard University, Cambridge, Massachusetts, USA; STFC Rutherford Appleton Laboratory, United Kingdom

**Keywords:** X-ray crystallography, scaling, variational inference, deep learning

## Abstract

X-ray diffraction enables the routine determination of the atomic structure of materials. Key to its success are data-processing algorithms that allow experimenters to determine the electron density of a sample from its diffraction pattern. Scaling, the estimation and correction of systematic errors in diffraction intensities, is an essential step in this process. These errors arise from sample heterogeneity, radiation damage, instrument limitations and other aspects of the experiment. New X-ray sources and sample-delivery methods, along with new experiments focused on changes in structure as a function of perturbations, have led to new demands on scaling algorithms. Classically, scaling algorithms use least-squares optimization to fit a model of common error sources to the observed diffraction intensities to force these intensities onto the same empirical scale. Recently, an alternative approach has been demonstrated which uses a Bayesian optimization method, variational inference, to simultaneously infer merged data along with corrections, or scale factors, for the systematic errors. Owing to its flexibility, this approach proves to be advantageous in certain scenarios. This perspective briefly reviews the history of scaling algorithms and contrasts them with variational inference. Finally, appropriate use cases are identified for the first such algorithm, *Careless*, guidance is offered on its use and some speculations are made about future variational scaling methods.

## Abstract 2

Emerging algorithms based on machine learning offer promise in processing new diffraction experiments.

## 1. Introduction

X-ray diffraction from crystalline samples is a widely used technique for determining the molecular properties of samples at atomic resolution. A key challenge in deducing the atomic structure from observed diffraction intensities is that these intensities need to be corrected for a range of physical effects due to sample imperfections, radiation damage, X-ray absorption, and source and detector characteristics. The process of correcting for such artifacts is known as *scaling*, and is essential to obtain high-quality experimental structures. With the advent of accurate structure prediction and determination methods, the focus of macromolecular diffraction experiments is shifting towards comparative experimental designs that emphasize, for example, the detection of ligand-binding events, as in high-throughput drug-fragment screens (Hartshorn *et al.*, 2005 ▸; Murray *et al.*, 2007 ▸), time-resolved studies using X-ray free-electron lasers and synchrotron beamlines (Tenboer *et al.*, 2014 ▸; Carrillo *et al.*, 2021 ▸), and the use of physical perturbations (Hekstra *et al.*, 2016 ▸; Thompson *et al.*, 2019 ▸). These studies seek to detect *differences in diffraction intensities* between data sets, placing stringent demands on the accuracy of corrections for systematic errors.

In this topical review, we provide a brief overview of diffraction experiments on crystalline samples and the factors that affect the final diffraction intensities, followed by a discussion of past and present scaling algorithms seeking to correct measured intensities. We then focus on a new class of scaling algorithms based on variational inference and deep learning. We conclude with a discussion of the design of a specific algorithm, *Careless*, and share our thoughts on best practices for its use, along with potential future developments and extensions.

## 2. Diffraction experiments

In a typical diffraction experiment, a crystalline sample is exposed to high-energy particles, usually X-ray photons, and the scattered particles are recorded on a two-dimensional detector. In the case of X-rays, their electric field component interacts strongly with the electrons in the sample. Some X-rays are scattered by these electrons and yield patterns on the detector that provide information about the positions of electrons, and thereby the atoms, in the sample.

**Figure 1:**
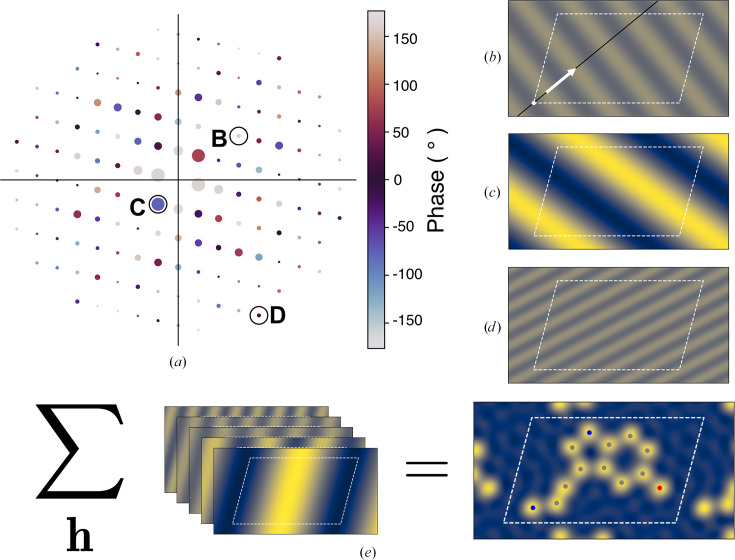
(*a*) Schematic of a diffraction pattern. The phase of each reflection is represented by color and its intensity is represented by the size of the reflection. Reflections B, C and D are highlighted with black circles. (*b*, *c*, *d*) The spatial frequency components of the electron density encoded by structure factors which correspond to the circled reflections B, C and D, respectively, in (*a*). The outline of the unit cell is shown as white dashes. Reflections farther from the origin exhibit a higher spatial frequency (for example reflection D). Brighter reflections correspond to higher-amplitude spatial frequency (for example reflection C). (*e*) Summing over all components results in the electron density of the sample.

Scattering from crystals is known as diffraction. Due to the repeating nature of crystals, scattered X-rays form discrete spots, or reflections, on the detector (Fig. 1 ▸ *a*) where the conditions are met for constructive interference between X-rays scattered from different unit cells, the repeating units of the crystal. Each reflection represents an interference maximum indexed by a triplet of integers called Miller indices. The reflections each have two associated parameters: amplitude and phase. Amplitude and phase dictate how much and in what manner a reflection contributes to the total electron density. The amplitudes are experimentally observable, while the phases are not directly observable but can be determined by certain experiments or, more frequently, computationally from a suitable starting model. The contribution made by a reflection is a plane wave (Figs. 1 ▸ *b*–1 ▸ *d*). The frequency and direction of the wave are calculated from the unit-cell constants of the crystal and the Miller index of the reflection. Summing the plane waves for every reflection yields the electron density of the sample (Fig. 1 ▸ *e*). The relative contribution of a particular reflection to this sum is given by the amplitude, which is proportional to the square root of the brightness (intensity) of the spot on the detector. Phase dictates the relationship of the spatial frequency vector of a wave (visualized as a white arrow in Fig. 1 ▸ *b*) to the origin of the crystal unit cell. Taken together, these quantities, amplitude and phase, are referred to as a *structure factor*.

**Figure 2:**
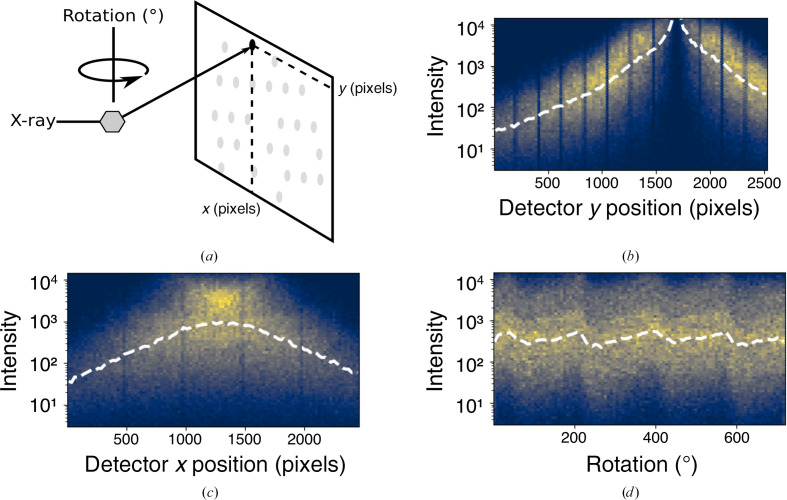
(*a*) Schematic representation of a rotation experiment. The crystal sample (shown as a gray hexagon) diffracts the incident X-ray beam. Diffracted X-rays are measured on a detector (shown as gray spots) at a particular *x*, *y* detector coordinate. (*b*, *c*, *d*) Two-dimensional histograms showing the distribution of measured reflection intensities for a hen egg-white lysozyme data set. The dashed white lines show the median intensity in each metadata bin. (*b*, *c*) The observed reflection intensities are dependent on the detector position. (*d*) The observed reflection intensities vary as a function of crystal rotation.

The squared amplitude is referred to as the intensity of a reflection. A central task in crystallography is to derive estimates for the intensity of each reflection. However, this task is complicated by physical factors that alter the measured intensity of a reflection based on its context. This phenomenon is best visualized by plotting the average intensity of reflections as a function of their context (Fig. 2 ▸). Ideally, the average intensity of the reflections should be uniform across the experiment. However, the observed intensity of the collected data depends nonlinearly on the positions of the reflections (Figs. 2 ▸ *b*–2 ▸ *d*). To faithfully estimate the intensity of the reflections, it is essential to estimate and correct for these artifacts.

## 3. Contributions to scale factors

The observed reflection intensities, *I*, on a diffraction pattern are proportional to the squared amplitude of the structure factors, *F*, of the crystallographic unit cell (Darwin, 1922 ▸), 

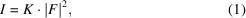

where *K* is the *scale factor*. Unfortunately, *K* varies from observation to observation. Under ideal experimental conditions, *K* depends on the brightness and shape of the incident beam, the wavelength and the crystal volume in the X-ray path. For polarized X-ray sources such as synchrotrons, the observed reflection intensity is also affected by the angle of the diffracted X-rays relative to the polarization of the incident beam. In conventional rotation-method experiments, the intensity further depends on the Lorentz factor, which accounts for the resolution-dependent speed at which the Bragg peaks traverse the diffracting condition. Detailed discussions of these contributions can be found in Holton & Frankel (2010 ▸) and Otwinowski *et al.* (2003 ▸).

In practice, additional *systematic errors* influence the scale factor *K*. These errors include crystal-related factors, such as lattice defects, radiation-induced damage or dehydration during exposure (Kabsch, 2010 ▸), absorption in the primary (incident) and secondary (scattered) beam directions by the crystal or surrounding material (Katayama, 1986 ▸), incident beam-intensity fluctuations (Evans, 2006 ▸) and instrument-related errors such as pixel and panel defects (Diederichs, 2010 ▸) or errors in integrated intensities due to errors in spot-position prediction. As a consequence, observed intensities for the same Miller index can rarely be compared directly, whether from the same crystal or not.

Successful electron-density reconstruction requires that symmetry-related and redundant observations be placed on the same scale. This procedure, known as *scaling*, is carried out by finding optimal scale factors *K* such that equation (1) provides a set of intensity values that agree as much as possible and can be properly compared. Parameterizing detailed physical models of scattering to determine *K* is difficult due to the many physical parameters that contribute to the scale in mathematically similar ways. Hence, despite recent efforts to model diffraction data from first-principles physics (Mendez *et al.*, 2020 ▸), most approaches still rely on an empirical routine to scale diffraction data.

## 4. Algorithms for scale-factor determination

The goal of a scaling algorithm is to place all symmetry-related and redundant reflections on the same scale. This is necessary for successful electron-density reconstruction and is critical when comparing data sets. Methodologically, reflection intensities are placed on a common scale by finding optimal inverse scale factors and subsequently dividing the observed reflection intensities by these factors. Scale factors are determined by optimizing an objective function which penalizes deviations between redundant reflection observations.

Let *I*_**h**,*i*_ denote the observed intensity corresponding to Miller index **h** = {*h*, *k*, *l*} on image *i*. Recall that the observed intensity is corrupted by various factors depending on the context of that image. We denote a corrected intensity as 

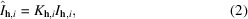

with corresponding uncertainty

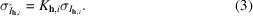




Estimates of the error in integrated intensities, 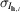 , are typically determined earlier during integration. The goal of scaling is to estimate optimal values of *K*_**h**,*i*_ such that merged intensities may be estimated by averaging, 

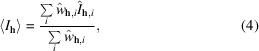

where the weights are typically the inverse variance of the corrected intensity introduced in equation (3), 

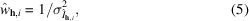

which is the maximum-likelihood weighting scheme under a normally distributed error model.

**Figure 3:**
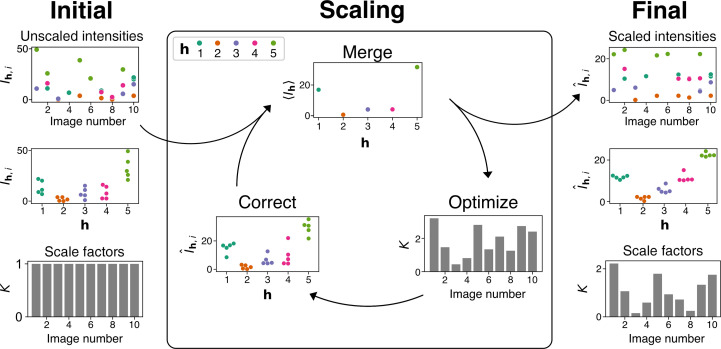
Schematic representation of a classical scaling algorithm. Scale factors, *K*, are initialized to *K* = 1 for each image and are iteratively optimized until convergence. The top left plot shows observed reflection intensity values on ten diffraction images; each color represents a distinct Miller index **h**. The middle left panel is a swarm plot of the observed reflection intensities (*I*_**h**,*i*_) for each Miller index **h**. Optimal scale factors are found by iteratively merging, optimizing *K* and correcting the unmerged reflections from the previous iteration. The algorithm iterates until the convergence of the objective function (equation 4) is achieved. The rightmost plots show the final values of the scaled intensities ( 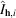 ) as a function of image number (top) or Miller indices (middle) and the optimal values of the scale factors *K* (bottom).

Most scaling algorithms work iteratively, estimating merged intensities and using optimization to find appropriate scale factors (Fig. 3 ▸).

The objective function for optimizing *K* typically takes the form of a least-squares model, introduced by Hamilton *et al.* (1965 ▸), 

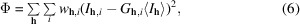

where 

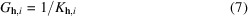

is the inverse scale factor. In this case, the weights, 

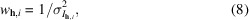

should be derived from the empirical, uncorrected uncertainties.

In Hamilton *et al.* (1965 ▸), the scale factor, *K*_**h**,*i*_, is parameterized by a single scalar value per image. By minimizing the objective function with respect to the scale factors, one will obtain a set of scale factors that correct redundant intensities to make them as close as possible to each other (Fig. 3 ▸). In this sense, the objective function serves as a measure of the performance of the model.

In essence, the objective function of Hamilton *et al.* (1965 ▸) remains in use in current scaling algorithms. However, modern scaling algorithms offer several important refinements of the core idea. For example, modern algorithms may include rounds of outlier rejection interspersed with merging and optimization to reduce the influence of spurious data points. In addition to the least-squares term, regularization terms may be added. Regularization terms generally penalize large differences in scale factors between observations, for example large higher order coefficients in absorption corrections using spherical harmonics (Evans, 2006 ▸) or large differences in per-image linear scale factors or *B* factors (Otwinowski *et al.*, 2003 ▸).

Most importantly, modern scaling algorithms provide more sophisticated parameterizations of the scale factors *K*. In the next section, we briefly cover the scale-factor parameterizations used by two popular scaling programs, *AIMLESS* and *XDS* (Kabsch, 2010 ▸; Evans & Murshudov, 2013 ▸).

### 4.1. Scale-factor parameterizations

Finding optimal scale parameters *K* via least-squares optimization of the objective function of Hamilton *et al.* (1965 ▸) (equation 6) requires the design of a parametric model for the scale factor and minimization of the objective function with respect to the scale-factor parameters. Scale-factor parameterizations depend on the mode of data collection. Historically, in macromolecular diffraction experiments the primary mode of data collection has been the rotation method (Arndt & Wonacott, 1977 ▸). In these experiments, a crystal is rotated at a fixed speed while X-ray exposures are collected (nearly) continuously, capturing the rotation of each reciprocal-lattice point through the Ewald sphere.

Various software suites include models designed to correct intensities originating from the rotation method. These software packages include *XDS* (Kabsch, 1988 ▸), *AIMLESS* (Evans & Murshudov, 2013 ▸), *HKL*-2000 (Otwinowski & Minor, 1997 ▸) and *DIALS* (Beilsten-Edmands *et al.*, 2020 ▸). Here, we briefly describe the parameterizations used by *XDS* and *AIMLESS*.

The scaling algorithm *AIMLESS* employs a physical model of the systematic errors that contribute to the observed intensity discrepancies. Specifically, the scale factor used in *AIMLESS* (Evans & Murshudov, 2013 ▸) accounts for radiation damage, absorption in the secondary-beam direction and a rotation-dependent scale factor. Attenuation due to X-ray damage is parameterized with a relative *B* factor as a function of rotation angle. Absorption in the secondary-beam direction is corrected by a scale factor determined by a sum of spherical harmonic basis functions. The rotation-dependent scale factor is parameterized as a smooth function of the rotation angle and it corrects for variations in the illumination volume or the absorption of the primary beam (Evans, 2006 ▸; Beilsten-Edmands *et al.*, 2020 ▸). Smoothness is enforced by representing each parameter by a set of prototypes spaced evenly across the rotation series. These prototypes are interpolated using a Gaussian kernel smoother (Murphy, 2012 ▸). Optimal scale factors are found through least-squares optimization. A scaling model used in *DIALS* is based on the physical model implemented in *AIMLESS* (Beilsten-Edmands *et al.*, 2020 ▸).

In contrast, the scale factor used in *XDS* utilizes a product of three two-dimensional functions to remove correlations between variations in reflection intensity and experimental parameters (Kabsch, 1988 ▸, 2010 ▸). Specifically, the scale factor accounts for radiation damage, absorption and non-uniform detector response, with each factor parameterized by a two-dimensional function. These functions take as input the image number and resolution, the *X* and *Y* location of reflections on the detector, and the image number and detector surface region, respectively. Unlike *AIMLESS* (Evans, 2006 ▸), *XDS* learns parameters on discrete grid points. Optimal scale functions are again found through least-squares optimization of the objective function of Hamilton *et al.* (1965 ▸).

Scaling algorithms tailored to the rotation method rely on prior knowledge of the experimental setup. Specifically, these algorithms assume an ordered set of diffraction images, with each subsequent image being related to the previous image by a small rotation. This approach relies on the intuition that systematic errors vary gradually across the data set.

### 4.2. Scaling methods for serial crystallography

Rotation-series scaling algorithms take as input ‘full’ reflection intensities, which are available because rotation sweeps the entire diffracting volume of each reflection. Advances in X-ray technology have led to the development of fourth-generation X-ray sources. The X-ray beams generated from X-ray free-electron lasers (XFELs), in particular, are much brighter than third-generation sources (Hattne *et al.*, 2014 ▸; Doniach, 2000 ▸). The femtosecond-duration XFEL-generated pulses can be focused to submicrometre widths, facilitating the use of microcrystalline samples, and are suitable for rapid-mixing experiments and photoexcitation studies (Liu *et al.*, 2013 ▸).

Moreover, the femtosecond durations of XFEL pulses allow one to collect diffraction data before the onset of radiation damage. However, focused XFEL pulses destroy crystalline samples after a diffraction event (Liu & Lee, 2019 ▸). Thus, to collect a full data set, thousands of microcrystals must be delivered to the beam in a serial manner. This mode of data collection is known as serial crystallogaphy (SX), or as serial femtosecond crystallography (SFX) when used with XFELs (Martin-Garcia, 2021 ▸). Unlike the rotation method, oscillating a crystal during diffraction is not possible, and each measured reflection is strictly partial. Additionally, each diffraction image generated in an SFX experiment originates from a single, randomly oriented microcrystal. These key differences require the estimation of full intensities for each reflection, and require indexing algorithms for randomly oriented crystals (Kabsch, 2014 ▸; Chapman *et al.*, 2011 ▸; Sauter, 2015 ▸; Brewster *et al.*, 2019 ▸). Several programs have been developed to process SFX data, including *CrystFEL* (White, 2019 ▸), *nXDS* (Kabsch, 2014 ▸) and *cctbx.xfel* (Brewster *et al.*, 2019 ▸). These packages contain scaling routines which are specifically designed for serial crystallography. The algorithms are similar in design to that of Hamilton *et al.* (1965 ▸) but modify the parameterization of *K* to account for the independent nature of each image. Subsequent to scaling, partialities can be inferred and corrected for in a process called post-refinement (White, 2014 ▸; Uervirojnangkoorn *et al.*, 2015 ▸; Kabsch, 2014 ▸). Intuitively, post-refinement works by updating the empirical experimental geometry to better model the observed intensities. Post-refinement is similar to scaling, but *K* is parameterized by a geometric model using the crystallographic unit cell, orientation and mosaic parameters of the crystal to incorporate information on the distance of each reciprocal-lattice point to the condition for maximal diffraction (the Ewald sphere).

## 5. Scaling diffraction data by variational inference

Recently, Dalton *et al.* (2022 ▸) introduced an alternative to sequential least-squares optimization and merging. The algorithm is similar in spirit to the classic algorithm of Hamilton *et al.* (1965 ▸), yet it is implemented using modern machine-learning principles. Functionally, the most important difference is that merging and scaling occur simultaneously as part of the same optimization routine. Key to doing so is variational inference (VI). VI is a Bayesian estimation technique in which data (observed intensities) are used to update prior beliefs (see below) to yield a posterior probability for model parameters (here the structure-factor amplitudes and the scale factors) conditional on the observed intensities. Exact determination of this posterior distribution is generally not tractable. Instead, in VI one proposes a general functional form for the posterior distribution and optimizes the parameters of this ‘surrogate posterior’, also known as a variational distribution. The formalism used by Dalton *et al.* (2022 ▸) allows the use of several forms of the surrogate posterior. In its current form, the structure-factor amplitudes, *F*_**h**_, are treated as statistically independent from each other and from the scale factors, and are described by a truncated normal distribution for each amplitude. That is, the posterior distribution over structure factor amplitudes and scale factors is approximated as 

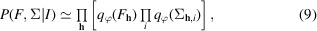

where Σ represents the scale factors and the surrogate posteriors are denoted *q*_φ_( ) with variational parameters φ. Somewhat similar to the scale functions in *XDS* and *AIMLESS*, the scale-factor function *q*(Σ) depends on metadata about each reflection (for example detector location, resolution and rotation angle). In the algorithm proposed by Dalton *et al.* (2022 ▸), however, the scale-factor function is parameterized by a neural network. This neural network takes as input user-specified metadata about each reflection, and calculates (a posterior distribution for) the scale factor for each reflection. This choice of scale-factor parameterization is innovative due to its generality. Neural networks are universal function approximators (Hornik *et al.*, 1989 ▸), meaning that the same inference routine can be applied to many types of diffraction data without the need to construct and calibrate detailed physical models of the experiment.

The algorithm then proceeds to estimate merged structure-factor amplitudes (along with the scale function) from unmerged reflection intensities by maximizing the following objective function (for a derivation and implementation details, see Dalton *et al.*, 2022 ▸), 

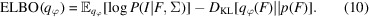




The Evidence Lower BOund (ELBO) is the standard objective function in VI (Blei *et al.*, 2017 ▸). To provide some intuition about the meaning of the ELBO, we observe that the first term, 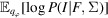 , is the expected log likelihood of the data set given the estimated values of the amplitudes and scale factors. This term ensures that the structure factors faithfully represent the experimental data. In general, the description of the likelihood function should reflect the understanding of experimental errors. The precise form of the error model used in variational inference is a modeling choice. However, the flexibility of VI allows options beyond the normally distributed error model implied in the historical least-squares objective function of Hamilton *et al.* (1965 ▸). Specifically, Dalton *et al.* (2022 ▸) introduced a robust error model which uses a Student’s *t*-distribution. This distribution has heavier tails than the normal distribution and thus is more tolerant of outlying intensities in the data set. The degree of tolerance is specified by the ‘degrees of freedom’ parameter of the *t*-distribution. In the limit of infinity, the *t*-distribution becomes equivalent to a normal distribution. For smaller values, it becomes increasingly heavy-tailed and consequently less sensitive to outliers.

Because outlier rejection has been a very successful tool in crystallographic data processing, we wish to note that the architecture of the model does not preclude such methods. In fact, *Careless* outputs the mean and standard deviation of the predicted intensity for each reflection. In the future, these results could readily be used as the basis for an outlier-rejection algorithm which discards reflections that cannot be accurately described by the model.

The second term in the ELBO, *D*_KL_[*q*_φ_(*F*)||*p*(*F*)], is the Kullback–Leibler (KL) divergence between the variational distribution and a prior distribution, which is a measure of the similarity of two distributions that is minimal (zero) when the two are identical. This term can be thought of as a regularization penalty which enforces the assertion that the structure-factor distribution should resemble the prior. The simplest prior is the Wilson distribution (Wilson, 1949 ▸), which reflects the assumption that the unit cell of a crystal consists of atoms that are sufficiently randomly distributed that the central limit theorem applies to the resulting complex structure factors. Use of the KL divergence relative to a prior reduces overfitting. Note that one could also propose a prior distribution for the scale function, Σ, but this is not current practice.

## 6. Careless

The algorithm just described is implemented as a command-line interface program named *Careless*, which is written in Python using the TensorFlow library (https://github.com/rs-station/careless). *Careless* supports conventional CPUs. However, it benefits from GPU accelerators, and potential users should consider gaining access to modern NVIDIA GPUs if they wish to perform extensive work with *Careless*.

### 6.1. Should I use *Careless*?

The performance of *Careless* relative to other software has only been established in a limited number of cases (Dalton *et al.*, 2022 ▸). Thus, we offer some guidance for potential *Careless* users. For standard rotation-method X-ray diffraction data, the difference in the quality of *Careless* output relative to conventional packages seems to be minimal. Users are welcome to try the program on their conventional data, but we do not claim that it will make a substantial difference. In contrast, for Laue crystallography, where the X-ray beam is polychromatic, we strongly encourage users to try *Careless*. *Careless* was designed to natively support Laue crystallo­graphy and provides wavelength normalization and harmonic deconvolution. To our knowledge, *Careless* is the only open-source package that provides these capabilities besides *LSCALE* from the Daresbury Laue Suite (Arzt *et al.*, 1999 ▸). In synchrotron and XFEL serial crystallography, we also recommend the use of *Careless* for modestly sized data sets. Dalton *et al.* (2022 ▸) demonstrated that the model performs well for such serial data, especially when layers with per-image parameters (image layers) are appended to the neural network. *Careless* performs well when all data fit within the memory of a single GPU accelerator (typically data sets of up to 10 000 images). Larger data sets require slower, CPU-backed training. In certain cases, we have found it worthwhile to train the model on CPUs despite the extended run times, but this is not a recommended practice.

### 6.2. Cross-validation for resolution determination

Analogous to conventional merging packages, *Careless* can provide estimates of half-data-set correlation coefficients, CC_1/2_. We recommend using this measure to determine the resolution cutoff. Because of the architecture of the model, calculating CC_1/2_ is more computationally expensive than for conventional merging. To estimate it, the scale function and structure factors are first trained on the full data set. Subsequently, the scale model is frozen and used to estimate structure factors separately for random half data sets. Because it requires additional training time, this functionality is disabled by default. The --merge-half-datasets flag in the command-line interface will enable half-data-set merges after initial model training. The output is saved to a special MTZ file, which can be analyzed with an included program, *careless.cchalf*. If error estimates of CC_1/2_ are desired, they can be obtained from repeated partitioning of the data set using the --half-dataset-repeats flag. We have found that the popular resolution cutoff of CC_1/2_ ≥ 0.3 works well for *Careless*.

### 6.3. Cross-validation of hyperparameters

*Careless* has several hyperparameters that may impact model performance. Selecting these hyperparameters wisely is crucial in order to obtain high-quality results. To help with this selection, *Careless* offers a form of cross-validation, which is uncommon in conventional scaling packages. Specifically, users can compute CC_pred_, the correlation coefficient between the intensities predicted by the model and the observed intensities. This is especially powerful when a fraction of the observations are withheld during model training using the --test-fraction flag. CC_pred_ can be calculated using *careless.ccpred*. Users can diagnose overfitting by looking at the gap between CC_pred_ for the test and training fractions, similar to the gap between *R*_work_ and *R*_free_ used in structure refinement. Functionally, hyperparameters should be chosen to maximize CC_pred_ for the test set.

### 6.4. Common hyperparameters

One of the strengths of *Careless* is its flexibility. As demonstrated by Dalton *et al.* (2022 ▸), *Careless* can model drastically different types of diffraction experiments. However, this flexibility also results in several hyperparameters that the user must adjust. The most important hyperparameters are summarized in Table 1 ▸ as *Careless* input flags. Here, we provide some advice about sensible default settings.

The first and most obvious hyperparameters are the identities of the metadata provided for each reflection. At a minimum, we believe these should include the detector coordinates of the reflection observation and the resolution of the reflection (‘dHKL’) or the *P*1 Miller indices (‘Hobs’, ‘Kobs’ and ‘Lobs’). These two sets of parameters allow the model to perform isotropic or anisotropic scaling, respectively. For single-crystal data, it also makes sense to incorporate some notion of image number or rotation information into the metadata. This allows the model to learn to inflate the scale factors observed later in the data set, which accounts for radiation damage. Different types of diffraction experiments may require additional metadata. For Laue experiments, it is essential to include the empirical wavelength of each reflection observation to enable wavelength normalization. Likewise, for serial crystallography data it is important to include an empirical estimate of the Ewald offset, which can be represented by a scalar or vector quantity. Further, regarding serial crystallography, we find that image layers have a dramatic impact on performance. However, including too many layers can lead to overfitting. If the compute budget permits, we recommend trying a series of layer counts --image-layers={1,2,4,8} and choosing the best performer based on CC_pred_. If compute time is limited we suggest using two layers, as this rarely results in overfitting. Anecdotally, image layers can also improve performance in single-crystal processing.

Another key hyperparameter is the degrees of freedom of the error model. By default, *Careless* employs a normally distributed error model. However, we have encountered many examples where it is advantageous to use the robust Student’s *t*-distributed error model. This model features a hyper­parameter, the degrees of freedom, which should be chosen by cross-validation for each data set. In our experiments, we typically explore a range of values in logarithmic spacing, for example {1, 4, 16, 64, 256, 1024}, and select the top performer by CC_pred_. However, we recognize that performing this sweep can be time-consuming. If you are resource-constrained, we recommend trying --studentt-likelihood-dof=32 in addition to the default normal error model. In our experience, this option rarely degrades performance and often provides improved results.

## 7. Future directions

One of the major benefits of the variational approach to X-ray data processing is its extensibility. We anticipate that over time, we and others will expand on the core model presented in Dalton *et al.* (2022 ▸) to further improve its accuracy and applicability. Below, we outline several anticipated extensions.

### 7.1. Error models

Although Dalton *et al.* (2022 ▸) demonstrated that Student’s *t*-distribution offers superior performance to a normally distributed error model in some cases, it may not be the optimal choice. For a positively distributed quantity such as intensity, the optimal error model may not be symmetric (see, for example, Greisman *et al.*, 2021 ▸). Future work may address this shortcoming by deriving a more appropriate error model.

### 7.2. Structured priors

In *Careless*, Wilson’s prior, based on a random-atom model, is applied independently to each structure factor. Modeling structure factors as statistically dependent could be advantageous. For instance, in comparative crystallography, multiple sets of closely related experiments are carried out simultaneously. In anomalous diffraction, the two halves of reciprocal space (Friedel mates) are closely related but contain small anomalous differences that can be used to localize atoms of certain elements or estimate phases. In upcoming work, we will describe how Friedel mates can be modeled as statistically dependent structure-factor pairs to recover more anomalous signal (for a preview, see the example *Using a bivariate prior to exploit correlations between Friedel mates* at https://github.com/rs-station/careless-examples/; see also Garcia-Bonete & Katona, 2019 ▸). For time-resolved crystallo­graphy experiments, modeling subsequent time points as statistically dependent could be advantageous. Similarly, when dealing with derivative structures resulting from isomorphous replacement or small-molecule ligand screens, one may want to scale a consensus set of structure factors against a native or apo data set, while modeling the perturbed (for example metal or ligand-soaked) crystals as dependent.

### 7.3. Stochastic training

Currently, a major limitation of variational scaling is that the entire data set must reside in memory alongside the model. Consequently, processing large serial crystallography data sets on contemporary accelerator cards is challenging. Our studies indicate that such data sets must be trained on a CPU node with a large amount of memory (512 GB in our case). Without GPU hardware acceleration, estimating structure factors for a serial data set with over 100 000 images can take weeks. One strategy to solve this problem is to parallelize the algorithm across multiple accelerators or even cluster nodes. However, our experiments indicate that this is not a viable strategy due to I/O overhead. A more effective approach is to adjust the model such that it can estimate accurate gradients for the structure factors from small batches of data. This method, known as stochastic training, is a popular paradigm in contemporary machine learning. Our preliminary work demonstrates that it is possible to reparameterize the scale model to accomplish this goal. In fact, this model can productively estimate global structure-factor gradients from as little as a single image at a time. A preliminary manuscript is available on the NeurIPS Machine Learning in Structural Biology Workshop webpage at https://www.mlsb.io/papers_2022/Online_Inference_of_Structure_Factor_Amplitudes_for_Serial_X_ray_Crystallography.pdf.

### 7.4. Direct pixel merging

The most exciting prospect for this technology is its direct application to diffraction patterns. Specifically, the form of the variational objective function does not require the calculation of the likelihood in terms of *integrated* reflection intensities. Instead, the likelihood function could be parameterized in terms of the pixels surrounding a Bragg peak. In this context, it would be natural to use a Poisson error model reflecting the uncertainty due to counting noise. This approach would require the incorporation of a model of the Bragg peak shape in the scale function. While *ad hoc* models such as a multivariate normal or Lorentzian lineshapes could be used, it is more appealing if the neural network could learn the suitable peak-shape model in a black-box manner.

## 8. Conclusion

Cutting-edge light sources pave the way for innovative experimental designs that enable a deeper understanding of protein dynamics through comparative experiments. As macromolecular crystallography shifts its focus to comparative experiments, accurate and reliable scaling methods become more crucial. New experimental designs using novel sample-delivery methods or novel experimental geometries may introduce new systematic errors. Traditional scaling algorithms often require the development of new packages to accommodate updated experimental designs. In contrast, the flexibility of machine-learning methods, as demonstrated by *Careless*, allows the generalization of data-processing algorithms. This adaptability enables researchers to quickly tackle new experimental methods without developing algorithms for each specific case. Moreover, these methods may be applicable to diffraction experiments involving neutron or electron beams. 

## Figures and Tables

**Table 1:** Flags used in *Careless*

Flag	Default	Description
--test-fraction	0.0	Fraction of the data held out for cross-validation
--merge-half-datasets	—	If True, merge pairs of random half data sets separately after training. Generates output files for estimating CC_1/2_.
--half-dataset-repeats	1	Number of half-data-set merging repeats. Used to estimate the uncertainty of CC_1/2_.
-d, --dmin	None	Set maximum resolution in Å. By default, merge reflections to the maximum input resolution.
--separate-files	False	Create an individual output file for each input MTZ. If True, data are scaled together and merged separately.
--studentt-likelihood-dof	None	Invoke Student’s *t*-distributed error model and set its degrees of freedom
--refine-uncertainties	—	Use the model in Evans (2011 ▸) to adjust the 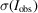
--positional-encoding-frequencies	4	Number of positional encoding (Zhong *et al.*, 2019 ▸; Mildenhall *et al.*, 2020 ▸) frequencies for metadata. Use with mlp-width to limit memory use. Default encodes all columns. For sepcific columns, see --positional-encoding-keys.
--positional-encoding-keys	None	Provide comma-separated metadata keys for positional encoding
--anomalous	—	Merge Friedel mates separately
--wilson-prior-b	None	Learn reflections on a specific global Wilson scale. The default Wilson prior is flat across all resolutions.
--mlp-layers	20	Number of neural network layers
--mlp-width	None	Width of hidden layers in the neural net; defaults to the dimensionality of the metadata array
--image-layers	0	Add additional layers with local, image-specific parameters
--disable-image-scales	—	Do not learn a scalar, per-image scale parameter
-w, --wavelength-key	Wavelength	For polychromatic data, the MTZ column name corresponding to reflections’ peak wavelength
